# Epigenetic signatures in cancer: proper controls, current challenges and the potential for clinical translation

**DOI:** 10.1186/s13073-021-00837-7

**Published:** 2021-02-10

**Authors:** Daniela Mancarella, Christoph Plass

**Affiliations:** 1 Division of Cancer Epigenomics, German Cancer Research Center (DKFZ), 69120 Heidelberg, Germany; 2 Faculty of Biosciences, Ruprecht-Karls-University of Heidelberg, 69120 Heidelberg, Germany; 3 German Consortium for Translational Cancer Research (DKTK), 69120 Heidelberg, Germany

**Keywords:** Epigenomics, Epigenetic signatures, Cell-of-origin, Cancer, Oncohistones, Tumor subclassification, Epigenetic therapy, Precision oncology

## Abstract

Epigenetic alterations are associated with normal biological processes such as aging or differentiation. Changes in global epigenetic signatures, together with genetic alterations, are driving events in several diseases including cancer. Comparative studies of cancer and healthy tissues found alterations in patterns of DNA methylation, histone posttranslational modifications, and changes in chromatin accessibility. Driven by sophisticated, next-generation sequencing-based technologies, recent studies discovered cancer epigenomes to be dominated by epigenetic patterns already present in the cell-of-origin, which transformed into a neoplastic cell. Tumor-specific epigenetic changes therefore need to be redefined and factors influencing epigenetic patterns need to be studied to unmask truly disease-specific alterations. The underlying mechanisms inducing cancer-associated epigenetic alterations are poorly understood. Studies of mutated epigenetic modifiers, enzymes that write, read, or edit epigenetic patterns, or mutated chromatin components, for example oncohistones, help to provide functional insights on how cancer epigenomes arise. In this review, we highlight the importance and define challenges of proper control tissues and cell populations to exploit cancer epigenomes. We summarize recent advances describing mechanisms leading to epigenetic changes in tumorigenesis and briefly discuss advances in investigating their translational potential.

## Background

DNA is organized in a packed structure within the nucleus. This structure is mediated by DNA associated proteins (called histones) and non-coding RNAs, which, together with the DNA, form a nucleoprotein complex referred to as chromatin. The entirety of chemical modifications of chromatin, referred to as “epigenome,” defines the state of chromatin and consequently gene transcription. One example for a modification of the chromatin is DNA methylation, a covalent conversion of cytosine to 5-methylcytosine preferentially found in the context of CpG dinucleotides. The interplay between epigenetic marks is highlighted by the observation that DNA methyltransferases (DNMT), the writers of DNA methylation, can be recruited by covalent posttranslational modifications (PTM) of histones. The chromatin state is not only influenced by modifications of the chromatin but also by the positioning and composition of chromatin components. Nucleosomes are octamers of histones around which the DNA is wrapped. The positioning of nucleosomes influences the accessibility of the DNA for transcription factors and thereby the chromatin state. Additionally, nucleosomes can differ in their composition of histones, also influencing chromatin states. Besides the canonical histones H2A, H2B, H3, and H4, which are expressed in a DNA synthesis-dependent manner, and the linker histone H1, non-canonical histone variants can be incorporated throughout the cell cycle affecting nucleosome stability and thereby chromatin accessibility for example [1]. Epigenetic features allow the creation of alternative phenotypes based on the same genetic information [1]. Cell types in an individual are defined by distinct, cell-type-specific epigenetic signatures. The process of differentiation and development is therefore associated with intense alterations of the epigenome [2, 3]. Alterations of the epigenome were additionally described in the processes of aging and senescence [4–6]. Besides alterations of the epigenome due to normal biological processes, aberrant modifications of the cell-specific epigenetic landscape can be found in association with diseases. Disruption of the epigenome on all levels, including nucleosome positioning associated with differential chromatin accessibility, histone PTMs, DNA methylation, and chromatin composition, is a molecular hallmark of cancer cells [7]. Cancer-associated epigenetic alterations can be reversed by epigenetic drug treatment and therefore present possible targets for cancer therapy [8, 9].

In this review, we discuss potential strategies and pitfalls in investigating cancer-specific epigenetics. We furthermore highlight the most recent achievements in cancer epigenetics, which are based on novel technologies with exquisite sensitivity in evaluating epigenomes with respect to the cell-of-origin. Additionally, we emphasize the translational implications of the most recent advances in epigenetic knowledge for cancer risk prediction, diagnosis, and therapy.

## Profiling of cancer epigenomes

Advances in sequencing-based technologies enable the identification of epigenetic signatures that have the potential to discriminate neoplastic from normal cells. This requires suitable controls, considering physiological variation of the epigenome mediated by aging and/or environmental stimuli. In addition, tumor heterogeneity and the cellular differentiation state of the cell populations analyzed must be considered since epigenetic heterogeneity has been described in solid tumors [10]. The need for reference epigenomes has been recognized by the International Human Epigenome Consortium (IHEC) [11] and the Encyclopedia of DNA Elements (ENCODE) Roadmap Epigenome Project [12]. Reference epigenomes from normal cells will provide a framework for epigenetic studies profiling cancer epigenomes [12].

### The reference epigenome

To define cancer-specific, aberrant epigenetic alterations, a comparison to a control epigenome, derived from those cells in which a transforming event originated, is necessary. This is not a trivial task since the normal epigenome can rapidly change in response to differentiation, aging, or microenvironmental stimuli. Age-associated epigenetic changes have been known for many years [13], but recent epigenomic profiling technologies allow for a more detailed analysis. Chromatin accessibility, as measured by nucleosome positioning, in B and T cells was shown to change with age and was connected to a reduction of canonical histones such as H3 and H4 over time. These alterations were associated, for example, with the silencing of the interleukin-7 receptor (*IL7R*) gene and further IL-7 signaling pathway genes [14]. Changes in DNA methylation, such as promotor hypermethylation, and histone modifications, such as decreased H3K4me3 and H3K27ac levels, were described during aging in human as well as in mouse hematopoietic stem cells. Affected genomic regions included genes involved in development- and cancer-associated pathways also found altered in leukemia. These alterations might explain the elevated cancer risk in older individuals [4, 6, 15, 16]. Increased age was also positively correlated with an increased instability of the chromatin state and hence, an increased heterogeneity of chromatin modifications was observed between older individuals in a twin study [5]. The comparison of histone PTMs in immune cells of young and older twins using mass cytometry analysis additionally highlighted a key role for environmental influences on chromatin marks [5]. Tobacco smoking was associated with DNA hypomethylation at CpG sites in the promoter or first exon of cancer-related genes as for example shown in breast cancer patients [17]. Furthermore, hypoxia was shown to decrease the activity of oxygen-dependent ten-eleven translocation (TET) enzymes which are responsible for DNA demethylation. The resulting DNA hypermethylation of promoter regions was associated with the promotion of tumor progression [18]. Together, these studies highlight the effect of physiological stimuli on the epigenome (Table 1). Knowledge and exclusion of physiologically induced epigenetic alterations is necessary to define disease-specific epigenetic alterations.

**Table 1:** Epigenetic signatures associated with specific physiological influences

Influence	Epigenetic signature	Affected processes	Samples used in the study	Reference
Environment	Increasing diversity of histone marks with age	-	Immune cells from young and old donors	Cheung, Vallania et al. 2018 [5]
Aging	Decreased chromatin accessibility at promoters and enhancers	T cell signaling	Immune cells young and old donors	Ucar, Marquez et al. 2017 [14]
Aging	Reduction of H3K27ac/H3Kme1/H3K4me3	Development, tumorigenesis	Hematopoietic stem cells young and old donors	Adelman, Huang et al. 2019 [15]
Aging	Broader H3K4me3 peaks, hypermethylation of TF binding sites	Differentiation, self-renewal	Young and old murine hematopoietic stem cells	Sun, Luo et al. 2014 [16]
Aging	Promoter hypermethylation	Stemness, tumorigenesis	Mouse colon derived organoids	Tao, Kang et al. 2019 [4]
Aging	Elevation of chromatin marks	-	Immune cells twins	Cheung, Vallania et al. 2018 [5]
Smoking	DNA hypomethylation in promoter regions	Tumorigenesis	Tumor tissue breast cancer patients	Conway, Edmiston et al. 2017 [17]
Metabolism	DNA hypermethylation	Survival, growth	Acute myeloid leukemia cells	Raffel, Falcone et al. 2017 [19]

### Cell-type heterogeneity

**Fig. 1:**
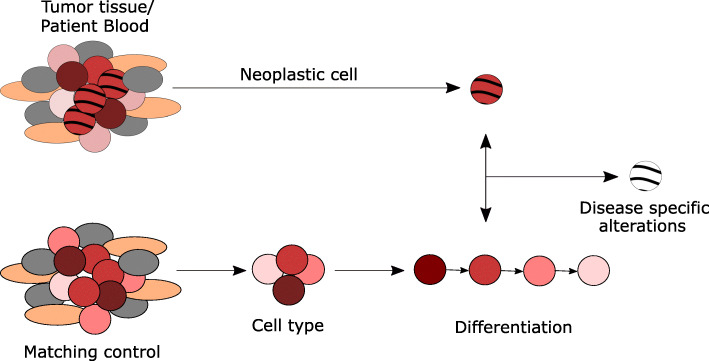
Reference epigenomes in cancer studies. Epigenetic studies use comparisons of normal and tumor samples to define disease-specific alterations. Even if those samples originate from the same patient (matching control, e.g., healthy tissue) and thereby exclude the detection of epigenetic variation based on environmental factors or aging, several other factors could potentially affect the analysis of disease-related epigenetic differences. The composition of cell types can differ between samples making the observed differences a mixture of cell-type-related and disease-specific divergences. The epigenome is also shaped during differentiation. Comparison of tumor and normal cells in different stages of differentiation would detect a mixture of differentiation- and disease-specific divergences. Therefore, factors affecting the normal epigenome have to be investigated and considered to define truly disease-specific epigenetic alterations

Comparison of DNA methylation patterns of different tissue samples showed high variation and distinct differentially methylated regions. Regions that varied the most between tissues were gene bodies, 3′-UTR, and sites that are not related to genes [20]. These variations depend on the epigenetic divergence between specialized cell types in tissues. Even the epigenomes of specialized cells from one tissue have different epigenetic signatures highlighting the diversity of epigenetic variability. For example, neuronal and non-neuronal brain cells can be distinguished by their chromatin accessibility [21]. Defined by ATAC-sequencing, open chromatin regions of neurons are more extensive, are located distal to transcription start sites, and show smaller overlapping regions with previously reported open chromatin from bulk brain tissue as compared to non-neuronal cells [21]. Histone PTMs also differ between cell types. The patterns of H3K9 dimethylation of gene coding regions and CpG islands can be used to distinguish monocytes from lymphocytes in human blood [22]. The main sources for epigenetic investigation of diseases are tissues and peripheral blood, which contain several different cell types, diseased and healthy, in varying proportions. Analysis of primary tumor tissues or blood samples therefore presents a mixture of cell-type- and possibly disease-related epigenetic signatures. This mixture may potentially lead to a misinterpretation of data. Early DNA methylation studies of leukemia, for example, were strongly influenced by cell composition differences of the peripheral blood of healthy donors and cancer patients [23, 24]. Data obtained from purified cell populations can serve as references to correct for potential cell-type heterogeneity and thereby disentangle disease-related epigenetic alterations. The first reference-based algorithm for cell-type deconvolution using DNA methylation as a surrogate for cell composition was developed by Houseman et al. [25]. Subsequently improved algorithms like EpiDISH, [26], RefFreeCellMix [27], MeDeCom [28], and BayesCCE [29] were developed. The latter three algorithms present reference-free methods that employ advanced machine learning approaches to infer cell-type compositions [30, 31]. Such algorithms were frequently applied in epigenomic studies for example in a study investigating potential DNA methylation biomarkers for early diagnosis of colorectal cancer (CRC) in blood [32]. Here, the Houseman algorithm [25] was used to estimate cell proportions of several leukocyte types and supported the identification of three differentially methylated regions able to distinguish CRC cases and controls [30]. Deconvolution techniques not only allow the estimation of cell-type proportions within blood or tissue but also enable DNA methylation-based analysis of immune cell infiltration. The ratio of neutrophils to lymphocytes, for example, was described as a prognostic marker for survival in breast cancer [33]. Immunomethylomics became particularly useful with increasing interest in immunotherapies and can be used to estimate the response to therapy [30]. Additional to deconvolution techniques, single cell epigenomics can help to overcome the limitations of bulk analysis. Single cell approaches enable the detection of rare subpopulations that cannot be found by bulk analysis. Single cell epigenetic techniques include DNA methylation analysis by reduced-representation bisulfite sequencing [34] or post-bisulfite adaptor tagging [35, 36]. In summary, the development of cell-type deconvolution techniques allowed exclusion of cell-type-dependent backgrounds in epigenetic studies and, together with single cell epigenomics, helped unravel cancer-specific alterations (Fig. 1).

### Identifying the cell-of-origin

Although confounding cell-type heterogeneity is now investigated as standard in epigenetic analysis, epigenomic programming during differentiation or aging effects are less frequently considered. Naïve embryonal stem cells (ESC) possess a mainly open chromatin conformation, globally low DNA methylation levels, and low levels of repressive histone marks such as H3K27me3 or H3K9me3 compared to primed ESC that cannot differentiate into every cell type anymore [2, 37]. Their epigenome further changes during terminal differentiation. It was estimated that one third of the entire epigenome changes during fibroblast differentiation for example [37]. Epigenetic alterations during differentiation have also been described in B cell maturation [3, 38], hematopoiesis [39], and osteogenic and adipogenic differentiation of mesenchymal stem cells [40]. By profiling of histone marks such as H3K27ac and chromatin accessibility, Rauch et al. described osteogenesis to be mainly driven by activation of enhancers preestablished in precursor cells [40]. Adipogenesis, in contrast, needs a remodeling of the entire chromatin landscape [40]. Using tagmentation-based whole genome bisulfide sequencing on DNA from highly selected B cell subpopulations in different maturation states, Oakes et al. demonstrated an unidirectional loss of DNA methylation mainly in promoter and enhancer regions during maturation. Furthermore, this study demonstrated that a maturation stage-specific epigenome is present in chronic lymphocytic leukemia (CLL) [38]. The comparison of B cells from healthy individuals and cancer patients therefore resulted in a mixture of disease and maturation-induced alterations. Understanding the epigenetic alterations during B cell maturation allows the separation of epigenetic alterations related to B cell programming from those that are disease-specific. As yet, data describing epigenetic changes during differentiation are rare for most cell types. Especially epigenetic alterations during differentiation of cells-of-origin of solid tumors, such as mesenchymal stem cells, have only recently been investigated [40]. Overall, epigenetics can unravel alterations in biological processes such as differentiation, which cannot be identified by genomic analysis. Epigenetic studies thereby allow a deeper understanding of cancer transformation. For instance, they can define cancer as a disease of impaired differentiation. Knowledge of alterations of the epigenome during differentiation is essential to unravel truly disease-specific epigenetic alterations (Fig. 1).

## Genomic aberrations inducing epigenetic alterations in cancer

Epigenetic alterations can be the result of increased mitotic activities of cancer cells. DNA hypomethylation of late replicating regions, with only a short time window for methylation of the daughter strand during S-phase, was for example associated to the mitotic age [41]. However, many tumors are associated with specific mutations in epigenetic modifiers or chromatin components. Studies of the effects of altered epigenetic modifiers and oncohistones, and thus, the mechanisms involved in establishing epigenetic alterations, allow to better understand the functional impact of specific epigenetic marks during oncogenic transformation. Exclusion of cell-type- or differentiation-related epigenetic alterations facilitates the analysis of disease-related effects of altered epigenetic enzymes or chromatin components on the epigenome.

### Altered epigenetic modifiers

**Fig. 2:**
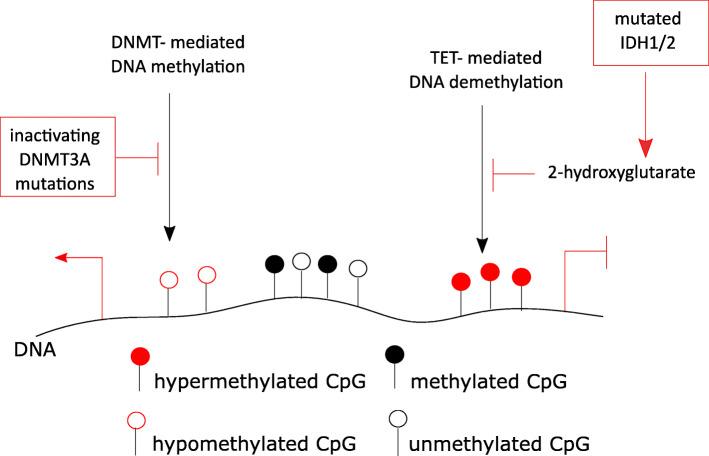
Effects of altered epigenetic modifiers. DNA methylation levels are maintained by a balance between methylation and demethylation. Mutations inactivating DNA methyltransferases (DNMT), such as DNMT3A, lead to hypomethylation and thereby affect gene transcription what might influence processes involved in tumorigenesis for example differentiation. Ten-eleven translocator (TET) enzymes demethylate DNA but can be inhibited by 2-hydroxyglutarate produced by mutated isocitrate-dehydrogenases. DNA hypermethylation is associated with gene silencing

Using suitable controls and genome-wide profiling, cancer-associated changes of DNA methylation, global hypomethylation, and focal hypermethylation were determined as early events in tumorigenesis. DNA hypermethylation in cancer, for instance, was found at CpG islands harboring both, the transcriptionally active histone mark H3K4me3 and the polycomb repressive complex-mediated transcriptionally repressive mark H3K27me3 [42]. These so-called bivalent regions, defined in embryonal stem cells, are associated with genes involved in apoptosis, DNA repair, or differentiation, processes known to be impaired in cancer. The epigenetic signature of a genomic region is thought to define its susceptibility to DNA methylation. Skvortsova et al. described a high ratio of H3K4me1/me3 as a predictor for promoter CpG hypermethylation in cancer [43]. This finding led to the suggestion that epigenetic signatures in a normal cell prior to transformation guide cancer-associated alterations. However, how cancer-associated epigenetic signatures are established is not well understood. Associations of cancer-specific, epigenetic signatures with genetic aberrations of epigenetic modifiers were described [44, 45]. Examples include mutations of the de novo *DNA methyltransferase 3a* (*DNMT3A*). *DNMT3A* was reported to be mutated in AML patients, and these mutations were described as driving events in hematopoetic malignancies [46]. The majority of *DNMT3A* mutations, including the highly recurrent R882H mutation, affect the catalytical domain and impair DNA methylation activity in a dominant negative way (Fig. 2) [47]. *DNMT3A* mutations were shown to lead to impaired differentiation of myeloid cells as well as reduced apoptosis [48]. Mutations of the counter players of DNMTs, the family of TET enzymes, that act as methylcytosine dioxygenases, were also reported in cancer [49]. Mutated TET1 or TET2 were suggested to induce global epigenetic alterations but whether their mutation alone is sufficient to establish an epigenetic cancer signature is unknown [7]. The mechanism by which mutated isocitrate-dehydrogenases 1 or 2 (IDH1/2) affect DNA methylation is well established. *IDH* mutants, as described in glioblastoma, produce the oncometabolite 2-hydroxyglutarate which inactivates DNA demethylating TET enzymes leading to DNA hypermethylation (Fig. 2) [49]. Mutated IDH1 alone was reported to be sufficient to alter the methylome genome-wide as shown in primary human astrocytes [50]. Further altered metabolic enzymes that affect TET activity and thereby DNA methylation include mutations of the succinate dehydrogenase complex subunit A (*SDHA*) [51] and an altered expression of the branched-chain amino acid transaminase 1 (*BCAT1*) [19]. Donaldson-Collier et al. investigated a direct link of a gain of function mutation in the H3K27 histone methyltransferase lysine-N-methyltransferase *enhancer of zeste homolog 2* (*EZH2*) on the epigenome. They found a globally increased level of H3K27me3 and subsequent transcriptional repression [52]. The repression was not randomly distributed within the genome but enriched in inactive topologically associated domains (TADs) with low expression of genes involved in proliferation [52]. TAD disruption associated with altered gene expression is a phenomenon also reported in cancer [53]. Epigenetic modifiers are not only affected by mutations but also by aberrant expression. The expression of *lysine-specific demethylase 4A* (*KDM4A*) was shown to be dysregulated in several tumors such as ovarian cancer [54]. Overexpression of *KDM4A* disrupted the equilibrium between KDMs and histone methyltransferases and facilitated DNA replication and thereby site-specific DNA copy number gains. Regions affected by amplification included pro-survival genes and oncogenes associated with drug resistance and poor outcome [54]. Expression of histone deacetylases (HDACs) was reported to be increased in several cancer types such as neuroblastoma and hepatocellular carcinomas [55]. The mode of action of HDAC dysregulation is diverse, as HDACs modify chromatin as well as non-chromatin components and thereby affect cell cycle progression, apoptosis, or DNA damage repair [55]. Overall, investigation of effects of altered epigenetic enzymes in cancer allows understanding the function of chromatin modifications on processes such as differentiation and cell cycle progression which are impaired in cancer. This knowledge may facilitate targeted drug development.

### Oncohistones

**Fig. 3:**
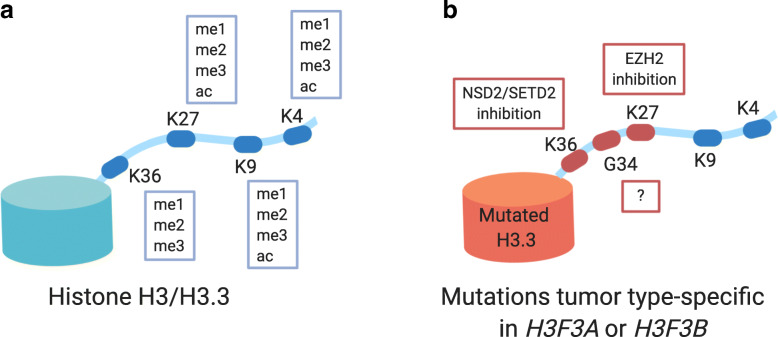
Impact of oncohistone mutations on modifications of the H3 and H3.3 N-terminal tail. **a** The N-terminal tail of H3 and H3.3 can be modified at several positions with different marks influencing the epigenetic state. K9me3 and K27me3 are associated with a heterochromatic state whereas K4me3, K36me3, and K27ac are found in active chromatin. **b** Some modification hot spots of H3 and H3.3 are found to be mutated in several cancer entities. They disrupt the epigenome by inhibition of epigenetic writers such as NSD2 or EZH2. The mechanism of H3.3 G34 mutation-associated disruption of the epigenome is currently intensely studied

The main protein components of chromatin, histones, were also found to be frequently mutated in cancer. Mutated histones are therefore often referred to as “oncohistones” [56]. Mutations affect all canonical histone families as well as non-canonical histones [56]. H2A and H2B were found mutated in carcinosarcoma whereas H1 mutations were found in diffuse large B cell lymphomas for example [57] [56]. Mutations in the canonical histone H3 and its non-canonical counterpart H3.3 were mainly found in pediatric tumors as for instance in high grade gliomas (HGG) of children [58, 59]. Although histones are encoded by several genes, mutations occur in a gene-specific manner and with defined mutations in certain tumor types suggesting a distinct cell-of-origin. Furthermore, oncohistone mutations occur in heterozygosity outlining a highly dominant effect [56]. Oncohistones were associated with global alterations of the epigenetic landscape. In brain tumors, a decrease of global levels of H3K27me2 and H3K27me3 and differential gene expression were linked to a H3.3 K27M mutation-dependent inhibition of EZH2 (Fig. 3) [58, 60]. Another mutation affecting a modification hotspot of histone H3 is the H3K36M mutation in chondrosarcoma which impairs K36 modifications by inhibition of the H3 lysine 36-specific histone methyltransferases *Nuclear Receptor Binding SET Domain Protein 2* (*NSD2*) and *SET domain containing 2* (*SETD2*) (Fig. 3) [61, 62]. Osteosarcoma and giant cell tumor of bone (GCTB) were shown to harbor H3.3 G34 mutations such as H3.3 G34W. These mutations do not affect a modification hotspot of histone H3.3 but were associated with global epigenetic alterations such as global hypomethylation [63, 64]. Voon et al. reported inhibition of histone demethylases leading to global increase in H3K36me3 and H3K9me3 levels due to expression of the mutant histone variant H3.3 G34R in mouse embryonal stem cells [65]. Studies in Hela cells could only find in *cis* effects of H3.3 G34 mutations, namely decreased H3K36me2 and me3 levels and increased levels of H3K27me3 [66]. How oncohistone-associated epigenetic changes induce tumorigenesis is currently under investigation. Larson et al. for example showed a selective effect of H3K27M-induced loss of H3K27me3 on transcription of bivalent genes involved in neuronal development in a mouse system for diffuse intrinsic pontine glioma (DIPG) [67]. Although oncohistone mutations were identified as initial oncogenic events as for instance shown by evolutionary reconstruction in DIPG biopsies [68], the role of oncohistones as tumor drivers is still under discussion. Several groups reported that oncohistone expression alone is not sufficient to induce tumor growth [67, 69, 70]. Additional genetic alterations, such as p53 mutations, or a dysregulated differentiation may be required to drive or promote tumorigenesis [68]. An impeded osteogenic differentiation was for example described for H3.3 G34W-expressing stromal cells in GCTB by Lutsik et al. [64]. Overall, the identification of oncohistone mutations by sequencing or immunohistochemistry improved diagnostics of certain tumors such as GCTB [71]. Additionally, oncohistone mutations provide opportunities to study the effects of histone modification, the interaction of histones with epigenetic modifiers, and their effect on biological processes which potentially induce tumorigenesis.

## Translational potential of epigenetic signatures

The identification of cancer-specific epigenetic signatures improves the quality of biomarker development based on epigenetic alterations. These distinctive cancer signatures that are independent of physiological influences can be used in precision medicine for diagnosis and clinical decision making and may allow the development of specific epigenetic drugs.

### Epigenetic signatures in cancer risk prediction, prognosis and diagnosis

Epigenetic alterations were reported to be induced by aging, chronic inflammation, and environmental stimuli such as cigarette smoking. These changes are stably transmitted to daughter cells even if a stimulus is not present anymore. Alterations accumulating in the epigenome can therefore be used as a bioarchive to predict cancer risks [72]. Age is a major cancer risk factor and alterations towards a cancer-like methylome may occur over time [6]. Yang et al. developed a model for a mitotic clock in normal and cancer tissues based on DNA hypermethylation that allows prediction of chronological age and mitotic activity (which reflects the number of cell divisions) that can be used to predict cancer risk [73]. DNA methylation analysis in combination with traditional risk prediction, by life history and mutational frequency, increased the prediction power for esophageal *cancer for example* [72]*.* In addition to risk prediction, diagnosis has also been improved by the analysis of epigenetic signatures. Some tumor entities are highly diverse, such as diffuse gliomas, and high interobserver variability in histopathological diagnosis was reported [74]. Molecular analysis, such as tests based on single-site DNA methylation, improved diagnosis and, subsequently, treatment [75]. Further development of such molecular diagnostics, for example, due to machine learning-based approaches, will help to refine diagnostics. A machine learning-based approach using genome-wide DNA methylation data developed by Capper et al. can be used to classify central nervous system tumors which standardizes diagnosis across centers [76]. The underlying hypothesis for the classification is that tumor subgroups carry the methylome of their cell-of-origin. In some cases, epigenetic subgroups within tumor entities were linked to different differentiation states of the cells-of-origin. Pilocytic astrocytomas, for instance, were classified into two subgroups with differential DNA methylation, particularly in developmental genes [77]. In colorectal cancer, methylation subgroups also reflect different differentiation states of the cell-of-origin [78]. The subgroup with a stem cell-like methylome signature, characteristic for an undifferentiated state of the cell-of-origin, was associated with reduced survival [78]. Methylome analyses were also shown to be able to distinguish subgroups of genetically homogenous tumor entities and allowed prediction of survival, risk of relapse or treatment outcome for cholangiocarcinoma [79], juvenile myelomonocytic leukemia (JMML) [80], atypical teratoid/rhabdoid tumors [81], brain tumors [82, 83], or lung tumors [84]. Global histone modification patterns analyzed by immunohistological staining or microarray approaches were shown to have a predictive power for cancer recurrence, for example, in prostate and bladder cancer [85–87]. DNA methylation analysis was able to discriminate lung cancers from head and neck metastasis and thereby provides support for the appropriate treatment choice [88]. DNA methylation-based cancer classifiers were also applied to determine the primary site of cancers of unknown primary and thus to improve treatment decisions and, potentially, outcome. Moran et al. described a diagnostic test based on microarray DNA methylation signatures established with a training set of around 3000 tumor samples with known origin. The test allows tumor type prediction with a specificity close to 100% [89]. Epigenetic approaches are also tested for non-invasive liquid biopsy approaches. Analysis of K27 trimethylated histone H3 in the blood of colorectal cancer patients by ELISA showed decreased amounts compared to healthy individuals [90]. Cell-free DNA (cfDNA) extracted from body fluids and analyzed for mutations or epigenetic modifications is another variant of liquid biopsies. So far, studies of cfDNA have relied on either mutational analysis of hotspot mutations or on personalized approaches after mutation identification in order to distinguish between normal and tumor-derived circulating DNA [91]. DNA methylation analysis of cfDNA allows a more universal assay design as the analysis can be restricted to CpG sites and does not require a scan of the entire genome. This decreases the amount of sample material required for analysis. Liang et al. showed targeted cfDNA methylation testing of only nine amplicons to be successful in detecting early-stage lung cancer in plasma [92]. In some cases, even the methylation status of a single CpG dinucleotide was demonstrated as a reliable biomarker. Methylation of a CpG dinucleotide 233 base pairs upstream of the transcription start site of *Zeta-chain-associated protein kinase 70* (*ZAP70*) for instance was reported as a marker for superior patient survival in CLL [75]. Furthermore, enrichment techniques for CpG sites using immunoprecipitation have the potential to increase the sensitivity of DNA methylation-based assays to make liquid biopsies suitable for treatment monitoring and investigation of minimal residual disease [91, 93]. Another advantage of DNA methylation analysis compared to transcriptional analysis is its suitability for the use with routinely collected formalin-fixed paraffin-embedded (FFPE) material. Although FFPE-induced DNA fragmentation inhibits PCR amplification, a robust PCR amplification of small DNA fragments can be assured by an increased polymerase concentration, dNTP concentration, and PCR elongation time [94]. DNA methylation patterns analyzed from glioblastoma FFPE material were shown to be predictive for patient survival [95]. In comparison to transcriptional analysis by immunohistochemistry or RNA Seq, DNA methylation analysis provides additional information about tumor-cell-intrinsic epigenetic regulation. An EZH2 deregulation found by DNA methylation-based footprinting analysis showed EZH2 as a potential therapeutic target in glioblastoma [95].

Overall, epigenetic alterations can be found across all types of cancer, making them attractive biomarkers (Table 2). Additionally, epigenetic changes appear early during tumorigenesis and are therefore suitable for early disease detection and intervention with epigenetic drugs [96]. Nonetheless, so far, only a few tests utilizing DNA methylation have received US Food and Drug Administration (FDA) approval. Examples include Cologuard and Epi proColon, both used for colorectal cancer screening [97]. As potential reasons for the small number of approved epigenetic-based tests, Koch et al. suggested various methodological retentions of epigenetic biomarker studies (“biased patient selection, improper study design and data analysis, lack of validation, and/or inappropriate reporting”). These retentions prevent the evaluation of the clinical value of epigenetic biomarkers and thereby their clinical translation [97].

**Table 2:** Epigenetics in cancer diagnosis and prognosis

Cancer	Epigenetic mark	Used for	Reference
Cancer of unknown primary	DNA methylation	Diagnosis	Moran, Martinez-Cardus et al. 2016 [89]
Central nervous system tumors	DNA methylation	Diagnosis, subclassification	Capper, Jones et al. 2018 [76]
Pilocytic astrocytomas	DNA methylation	Diagnosis, subclassification	Lambert, Witt et al. 2013 [77]
Colorectal cancer	DNA methylation	Diagnosis, subclassification	Bormann, Rodriguez-Paredes et al. 2018 [78]
Atypical teratoid/ rhabdoid tumors	DNA methylation	Subclassification	Johann, Erkek et al. 2016 [81]
Juvenile myelomonocytic leukemia	DNA methylation	Subclassification, clinical outcome prediction	Lipka, Witte et al. 2017 [80]
Cholangiocarcinoma	DNA methylation	Prognosis prediction	Goeppert, Toth et al. 2019 [79]
Brain tumors	DNA methylation	Disease progression and recurrence risk prediction	Sahm, Schrimpf et al. 2017 [82]
Brain tumors	DNA methylation	Survival prediction	Wiestler, Capper et al. 2014 [83]
Bladder cancer	Histone modification	Recurrence risk prediction	Ellinger, Schneider et al. 2016 [86]
Prostate cancer	Histone modification	Recurrence risk prediction	Seligson, Horvath et al. 2005 [85]
Esophageal cancer	DNA methylation	Risk prediction	Takeshima and Ushijima 2019 [72]
Colorectal cancer	Histone modification	Liquid biopsies	Gezer, Yoruker et al. 2015 [90]
Lung cancer	DNA methylation of cell-free DNA	Liquid biopsies	Liang, Zhao et al. 2019 [92]

### Epigenetic signatures and cancer therapy

The reversible character of altered epigenetic marks in cancer cells makes the epigenome a promising target for cancer therapies. Inhibitors of DNMTs and HDACs have been shown to be highly effective anticancer drugs due to their ability to inhibit tumor growth, reactivate silenced genes, and restore normal gene expression [9]. Therapies targeting the epigenome have been approved by the FDA. DNMT inhibitors, for example, have been approved for the treatment of older AML patients, and HDAC inhibitors have been approved for the therapy of cutaneous T cell lymphoma. The growth inhibitory effect of DNMT inhibition was shown to be associated with reduced DNA replication due to deactivation of replication origins potentially driven by the activation of tumor suppressor genes [98]. A combination of epigenetic drugs targeting the interplay between different epigenetic marks is recently studied as for instance the combination of EZH2 inhibition with drugs that impair H3K27 acetylation [99]. An increase in the histone mark H3K27ac after EZH2 inhibition was described to counteract the effect of EZH2 inhibition and was associated to *mixed-lineage leukemia 1 (MLL1*) expression. A combinatorial treatment sensitized resistant EZH2-overexpressing tumor cells to EZH2 inhibition. Patients with EZH2 overexpression could be stratified into EZH2 inhibitor mono- or combinatorial therapy in accordance with their MLL1 status [99]. Another approach is a combinatorial treatment with DNMT inhibitors (reduction of DNA methylation) and HDAC inhibitors (increase of transcriptionally active histone marks) studied in clinical trials [100]. Preclinical studies investigated the mode of action of the combinatorial treatment [101]. A synergistic effect predominantly on transcription of cryptic transcripts from normally inactive transcription start sites in long terminal repeat elements was described rather than an effect on the expression of canonical genes. The cryptic transcripts were thought to have potential immunogenic functions [101]. Inhibition of the *lysine-specific demethylase 1* (*LSD1*) (which increases H3K4me2 levels) was shown to induce the expression of double-stranded RNA from endogenous retroviral elements with potential to trigger antiviral responses. Due to these effects, LSD1 inhibition was shown to overcome resistance to checkpoint inhibition highlighting the potential of epigenetic drug treatment to improve immunotherapy [102]. A more targeted immunotherapy approach, utilizing the effects of an epigenetic mutation, is currently tested for diffuse midline gliomas. Patient-derived H3K27M mutant glioma cells were shown to have high expression levels of the disialoganglioside GD2 and an anti-GD2 CAR T-cell-mediated therapy was developed and is currently investigated [103]. Additionally, H3K27M itself was described as a neoepitope in gliomas and peptide vaccination showed promising results in mice studies [104]. These approaches specifically target tumor cells and thereby stand in contrast to epigenetic drug treatment with DNMT inhibitors for example that potentially affect neoplastic as well as normal cells. The development of more specific drugs selectively targeting mutated epigenetic modifiers will restrict effects to tumor cells only and decrease potential side effects. As an example, the inhibition of mutated IDH2 by small molecules is tested in clinical trials, and two inhibitors received FDA approval [105, 106]. Taken together, describing disease-specific epigenetic alterations allows the development of a variety of epigenetic drug-based treatment approaches for example for cancer patients.

## Conclusion and future directions

The challenge of cancer epigenetic studies is to define alterations induced by disease mechanisms as opposed to those preexisting in the cell-of-origin that undergoes tumorigenesis. Matching normal controls, considering patient-related information as well as cell types of the analyzed cell populations, improved the identification of disease-specific epigenetic alterations and are provided by consortia like the International Human Epigenome Consortium (IHEC). Knowledge of epigenetic alterations in differentiation also added to the improved description of cancer-related epigenetic alterations. But further research evaluating epigenetic alterations that occur in normal biological processes in more cell types is necessary to improve epigenetic studies for all cancer types. Epigenetic studies furthermore benefited from bioinformatic deconvolution approaches such as the Houseman algorithm. Using DNA methylation data, deconvolution techniques reduce cell-type- and composition-dependent backgrounds in studies of primary tissue and blood. Single cell approaches will further support epigenetic studies. A strict definition of disease-related epigenetic signatures allows the application of epigenetic alterations as biomarkers for precision medicine. Together with the knowledge of the cell-of-origin, disease-specific epigenetic signatures can support subgroup classification of cancers to improve diagnostics and prognostics. Furthermore, the definition of cancer-specific epigenetic alterations facilitates studies of mechanisms shaping cancer epigenomes. Disease-specific signatures can be linked to mutated epigenetic modifiers such as DNTMs that present potential therapeutic targets. Current studies investigate the mode of action of epigenetic drugs and develop drugs selectively targeting mutated epigenetic modifiers to improve therapy. All in all, overcoming the current challenges in epigenetic research will allow the discrimination of disease-specific and normal epigenetic alterations and thereby improve diagnostics, prognostics and therapies. Subsequent integration of epigenomic and genomic data will also aid clinical translation and may guide treatment for cancer patients.

## Data Availability

Not applicable.

## Abbreviations

AML: Acute myeloid leukemia
BCAT: Branched chain amino acid transaminase 1
cfDNA: Cell-free DNA
CLL: Chronic lymphocytic leukemia
CRC: Colorectal cancer
DNMT: DNA methyltransferases
ESC: Embryonal stem cells
EZH2: Enhancer of zeste homolog 2
FFPE: Formalin-fixed paraffin-embedded
GCTB: Giant cell tumor of bone
HDAC: Histone deacetylases
IDH: Isocitrate-dehydrogenase
KDM: Lysine-specific demethylase
LSD1: Lysine-specific demethylase
MLL1: *Mixed lineage leukemia 1*
NSD2: Nuclear receptor binding SET domain protein 2
PTM: Posttranslational modifications
SETD2: SET domain containing
TAD: Topologically associated domains
TET: Ten-eleven translocation enzymes
